# Subclinical Visuospatial Impairment in Parkinson’s Disease: The Role of Basal Ganglia and Limbic System

**DOI:** 10.3389/fneur.2014.00152

**Published:** 2014-08-11

**Authors:** Stefano Caproni, Marco Muti, Antonio Di Renzo, Massimo Principi, Nevia Caputo, Paolo Calabresi, Nicola Tambasco

**Affiliations:** ^1^Clinica Neurologica, Azienda Ospedaliera – Università di Perugia, Italy; ^2^Servizio di Fisica Sanitaria, Azienda Ospedaliera di Terni, Italy; ^3^Servizio di Neuroradiologia, Azienda Ospedaliera di Terni, Italy; ^4^I.R.C.C.S. – Fondazione S. Lucia – Roma, Italy

**Keywords:** Parkinson’s disease, fMRI, visuospatial/cognitive impairment, hippocampus, insula

## Abstract

**Background:** Visual perception deficits are a recurrent manifestation in Parkinson’s disease (PD). Recently, structural abnormalities of fronto-parietal areas and subcortical regions, implicated in visual stimuli analysis, have been observed in PD patients with cognitive decline and visual hallucinations. The aim of the present study was to investigate the salient aspects of visual perception in cognitively unimpaired PD patients.

**Methods:** Eleven right-handed non-demented right-sided onset PD patients without visuospatial impairment or hallucinations and 11 healthy controls were studied with functional magnetic resonance imaging while performing a specific visuoperceptual/visuospatial paradigm that allowed to highlight the specific process underlying visuospatial judgment.

**Results:** Significant changes in both cortical areas and subcortical regions involved in visual stimuli processing were observed. In particular, PD patients showed a reduced activation for the right insula, left putamen, bilateral caudate, and right hippocampus, as well as an over-activation of the right dorso-lateral prefrontal and of the posterior parietal cortices, particularly in the right hemisphere.

**Conclusions:** We found that both loss of efficiency and compensatory mechanisms occur in PD patients, providing further insight into the pathophysiological role of the functional alterations of basal ganglia and limbic structures in the impairment of visuoperceptual and visuospatial functions observed in PD.

## Introduction

Cognitive impairment is common in Parkinson’s disease (PD), even in the early stages, affecting around 25% of patients without dementia at the time of the diagnosis (1). Cognitive changes are mainly characterized by executive, memory, and visual perception deficits (2).

In PD patients, visuospatial deficits have been identified using tests of line orientation, memory for spatial location, and mental rotation. Furthermore, impairment in visuoperceptual abilities has been observed in object detection, categorization of visual stimuli, and face recognition (3). It has been hypothesized that these alterations play a role in the mechanisms of pivotal motor signs of the disease, such as freezing of gait (4, 5), and are connected to the development of visual hallucinations (1, 6). Recent findings have also suggested a role for lateralization of the basal ganglia circuits in stimuli perception, which determines different clinical manifestation in left- and right-side PD onset (7).

Over the last few years, visual perception and its underlying mechanisms have been investigated in PD, with increasing interest in the relationship between cognitive abilities and structural neuroradiological substrates (2, 8–10). However, only few studies have taken into account functional cortical activation of areas and networks involved in the visual perception processes, especially in the early stages of PD. Thus, this study aimed to detect brain activation during a specific visuoperceptual/visuospatial task in cognitively unimpaired PD patients, compared to healthy controls, using functional magnetic resonance imaging (fMRI), in order to investigate the salient aspects of the subclinical impairment of the visual perception network.

## Materials and Methods

### Patients

Eleven right-handed non-demented right-sided onset PD patients (8 males, 3 females; mean age: 65 years, range: 59–75), mean disease duration 3.8 ± standard deviation 1.5 years [mean Unified Parkinson’s Disease Rating Scale (UPDRS) off-state: 20 ± 4.5; mean Hoehn and Yahr scale (11): 2, range 1–3; mean Mini-Mental State Examination (12): 27.1 ± 1.4], all treated with levodopa (mean dose: 500 ± 100 mg daily) were recruited. Inclusion criteria were: clinical diagnosis of PD according to the United Kingdom Parkinson’s Disease Society Brain Bank criteria (13); no evidence of dementia according to both DSM-IV criteria and clinical diagnostic criteria for dementia associated with PD, published by Emre et al. in 2007 (particularly with normal attention, executive, and visuospatial features) (14); the absence of hallucinations; no evidence of depression symptoms at the assessment using the Beck Depression Inventory (15); no significant vascular damage, no brain tumors, no marked cortical, and/or subcortical atrophy on MRI scan; no use of anxiolytics, antidepressants, or antipsychotics, which could potentially affect cerebral blood flow; an optimal motor performance and no excessive movement artifacts during fMRI.

Eleven healthy right-handed age and sex-matched subjects served as controls. None of the control subjects had past or present neurological, cardiovascular, or psychiatric diseases. All participants gave their written informed consent to the study, which was approved by the local Ethics Committee (Umbria CEAS), according to the principles expressed in the Declaration of Helsinki. Complete demographic characteristics of the PD patients and controls are listed in Table 1.

**Table 1:** **Clinical and demographic characteristics of patients and control subjects**.

	Sex	Age	PD duration	Hohen and Yahr	UPDRS off-state	MMSE	LEDD (mg)
**PD PATIENTS**							
1	m	59	3	1.5	14	28	450
2	m	75	3	2	19	27	450
3	m	58	2	1	19	30	450
4	m	64	3	2	19	27	450
5	m	64	5	2	14	28	500
6	m	65	5	2.5	25	26	500
7	m	67	4	2	24	26	500
8	m	69	5	3	30	26	500
9	f	60	5	2	18	27	500
10	f	65	2	1	8	28	400
11	f	69	5	3	30	25	800
Mean		65	3.8	2	20	27.1	500
**CONTROLS**							
1	f	60					
2	m	76					
3	f	56					
4	m	68					
5	m	63					
6	m	63					
7	m	66					
8	m	71					
9	m	59					
10	m	64					
11	f	70					
Mean		65.1					

*UPDRS, Unified Parkinson’s Disease Rating Scale; MMSE, Mini-Mental State Examination; LEDD: Levodopa equivalent daily dose*.

### Visuoperceptual/visuospatial task

**Figure 1:**
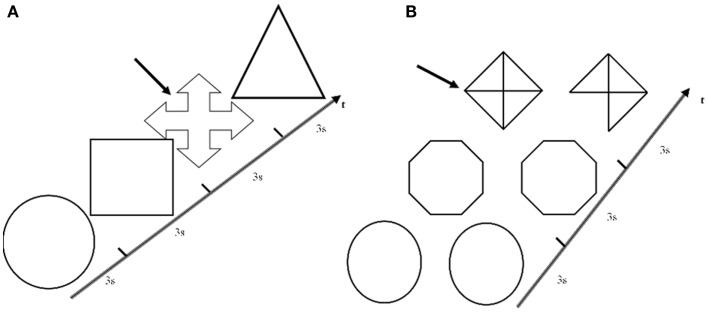
**Visuoperceptual and visuospatial tasks. (A)** An example of a figure sequence presented in the visuoperceptual task. Subjects were asked to press the key when the target image indicated by the arrow appeared. **(B)** An example of a figure sequence presented in the visuospatial task. Subjects were asked to press the key when coupled figures resulted different (as indicated by the arrow). For both tasks, seven slices lasting 3 s were presented during each epoch.

The subjects laid in the scanner and could read the instructions displayed on a white panel placed in the front of the scanner. A right-sided keyboard was connected to the magnetic resonance console to enable the observers to monitor the whole test. All subjects performed a structured paradigm organized according to a classic block design having two conditions. Abstract geometric meaningless images were used for both tasks. In the visuoperceptual task (VP), a sequence of diverse single figures were projected on the panel, and the subjects were asked to press the key with the index finger when a previously defined image appeared (Figure 1). This target image was definitely different from all the others, in order to minimize the memory and visuospatial effort. In the visuospatial task (VS), a sequence of coupled figures were projected, which were identical or partially different (orientation, lack of a piece, partial filling, …). The subjects had to press the key when coupled figures were different (Figure 1). The subjects were instructed to practice both tasks just before the fMRI scanning, to avoid over-training and mnemonic learning of the image sequences. All exams were performed at the same hour (3:00 or 4:00 p.m., in off-state, at least 12 h after the last administration of l-dopa). Data acquisition for the entire paradigm was obtained during a single MRI.

### MRI data acquisition

In this study, a 1.5 T Philips scanner was used, equipped with whole-brain single-shot 3D blood oxygen level dependent echoplanar imaging (EPI) hardware. Head pads and a firm chin strap immobilized head flexion-extension. Thirty-four axial slices of 4 mm thickness, parallel to the intercommisural plane (from *z* = −50 mm to *z* = + 80 mm), were collected using an EPI gradient echo sequence: echo time = 50 ms; repetition time (TR) = 3000 ms; flip angle = 90°; field of view = 230 mm; voxel size = 3.59 × 3.59 × 4 mm^3^; matrix = 64 × 64. T1-weighted images were also acquired. Data acquisition was organized in an epoch-related design. Acquisition time was divided into VP periods followed by VS periods. Each period consisted of seven EPI acquisitions of 3000 ms (TR) each, 21 s in total. For both VP and VS, the image’s projection lasted for a whole EPI acquisition (3000 ms), resulting in seven images per epoch. The two exercises were performed for 8 periods, for a total of 16 periods, divided into 112 volumes. Tasks lasted 336 s, corresponding to 5 min and 36 s. Potential brain abnormalities were previously excluded by examining conventional FLAIR, T2-weighted, and T1-weighted images.

### MRI analysis

Functional magnetic resonance imaging data were analyzed using SPM5 (Statistical Parametric Mapping, Wellcome Department of Cognitive Neurology, London, UK) (16). The functional images were co-registered and realigned to the first volume to correct for head translation or rotation during the scanning and to avoid incorrect spatial coordinates of activated voxels. Images were also normalized, using a standard voxel size 2 × 2 × 2 mm^3^, to the stereotaxic space of Talairach and Tournoux (17) using the three-dimensional volume (18). Images were also spatially smoothed with a Gaussian kernel of 8 mm full-width half maximum and temporally smoothed with a Gaussian kernel (FWHM = 8 s) (19). Statistical analysis of the activations obtained during the performance of tasks was based upon an epoch-related experimental design. The data obtained were modeled with a hemodynamic response function having an impulsive local flux variation. The sum of hemodynamic variations during an active period allowed for the calculation of a mean cortical activation during an exercise performance, obtained by applying the General Linear Model, *y* = (β/βerr)*x + *c* (16). Therein, the whole-brain mean activation signal corresponding to VS was compared to VP, by performing a first-level fixed-effect analysis having a cluster threshold of 10 voxels with *p* < 0.001 for all subjects. Group data were obtained through a random-effect second-level analysis using the SPM5 software package and were used to calculate a between-group analysis. The second-level analysis provided results on over-activations of whole-brain cortical areas and subcortical regions, with intensity measured by *F*-score. In summary, the final contrast revealed: PD patients (VS > VP) versus Controls (VS > VP).

## Results

### Behavioral results

All subjects correctly carried out both tasks. Both PD patients and control subjects made a similar number of errors for both tasks that did not differ significantly (*p* > 0.05). None of the subjects performed any visible movements other than those required by the tasks.

### fMRI results

**Figure 2:**
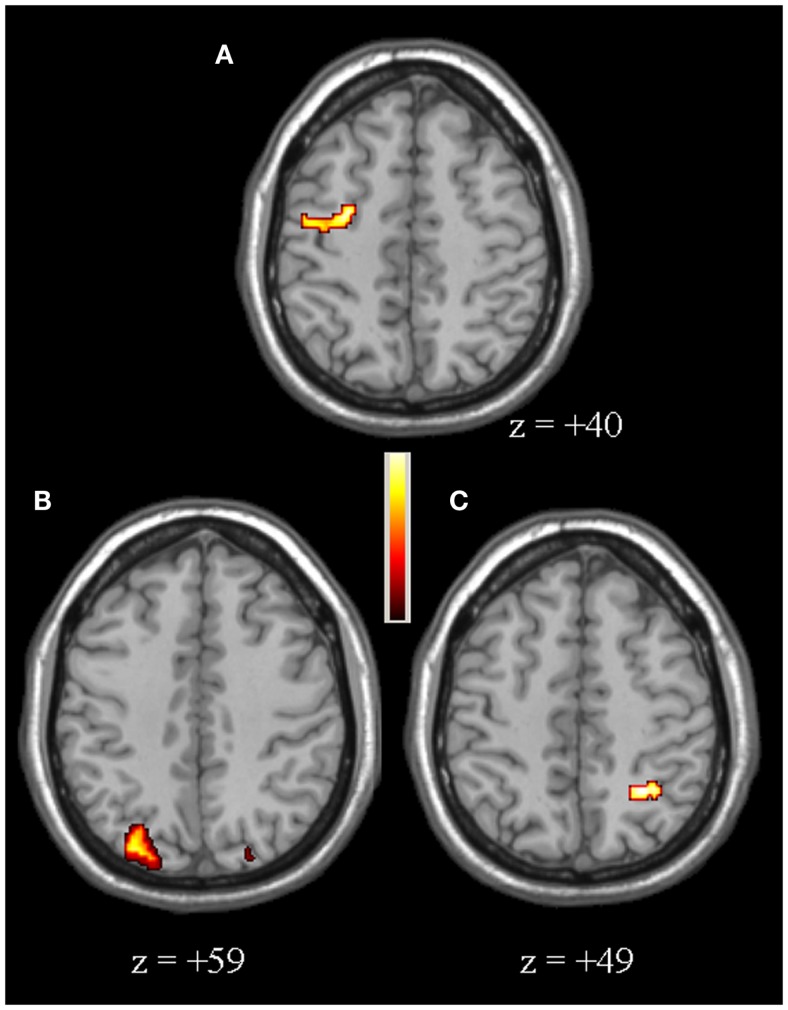
**[PD patients (VS > VP)] > [Controls (VS > VP)]**. The over-activations of right dorso-lateral-prefrontal cortex **(A)**, right **(B)**, and left **(C)** posterior parietal cortex observed in patients, compared to controls, for the second-level analysis VS > VP are shown. Colors bar range for *F*-score: 2–17.95.

**Figure 3:**
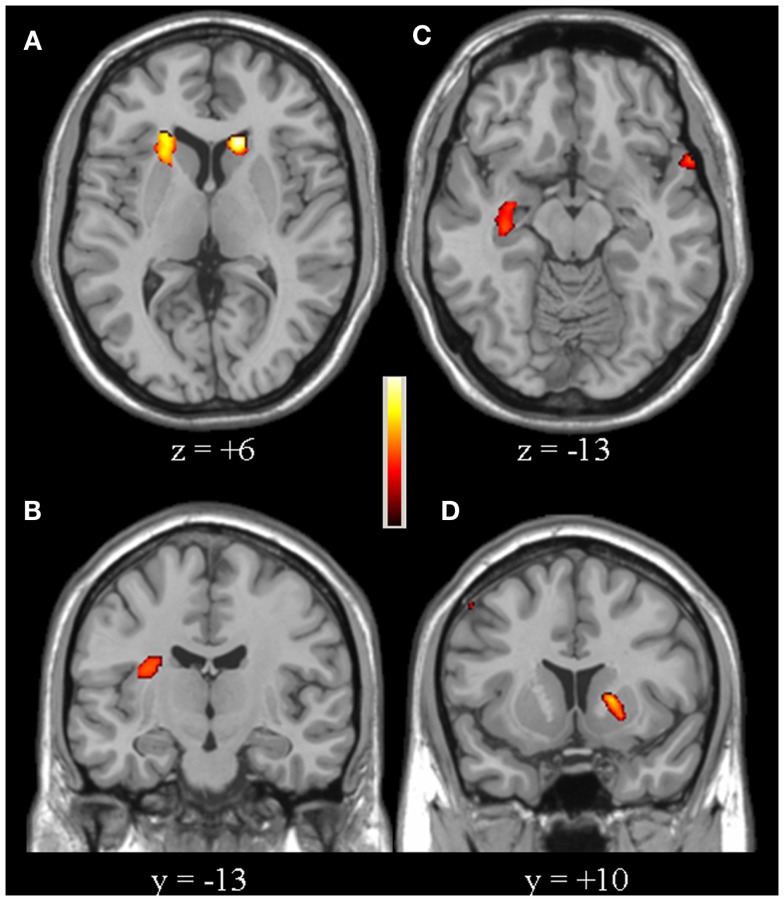
**[Controls (VS > VP)] > [PD patients (VS > VP)]**. The over-activations of bilateral caudate **(A)**, right insula **(B)**, right hippocampus **(C)**, and left putamen **(D)** observed in controls, compared to patients, for the second-level analysis VS > VP are shown. Colors bar range for *F*-score: 2–16.78.

Compared to controls, for the second-level between-group comparison VS > VP PD patients had an over-activation of the right dorso-lateral prefrontal cortex (DLPFC), defined as the sum of part of Broadman areas 9 and 46, and bilateral posterior parietal cortex (PPC), defined as the sum of Broadman areas 7 and 40 (particularly in the right hemisphere) (Figure 2, Table 2). Whereas, controls had a greater activation of the right insula, left putamen, bilateral caudate (particularly in the head), and right hippocampus (Figure 3, Table 2).

**Table 2:** **VS > VP second-level analysis results**.

Comparison	Area	Coordinates (mm)			*F*-score
		*x*	*y*	*z*	
PD > Controls	R DLPFC	−38	7	40	17.95
	L PPC	32	−59	49	12.51
	R PPC	−19	−69	59	14.75
Controls > PD	R insula	−34	−14	20	12.20
	L putamen	20	10	6	16.78
	L caudate	14	22	6	11.83
	R caudate	−18	24	6	16.67
	R hippocampus	−35	−15	−13	14.26

*For each region, Talairach coordinates and *F*-scores of the local maxima are reported (*p* < 0.001). DLPFC: dorso-lateral prefrontal cortex; L: left; PPC: posterior parietal cortex; PD: Parkinson’s disease; R: right*.

## Discussion

This is the first fMRI study investigating specific subclinical VS/VP aspects in cognitively unimpaired PD patients without hallucinations, providing evidence on the functional activity associated with visual perception in PD.

In this study, we used a specific paradigm that allowed us to differentiate the most important functions of visual perception in the same fMRI exam. Visual perception is divided into two functionally and neuroanatomically distinct systems: the visuoperceptual or “what” system, encoded by the “ventral” occipito-temporal pathway, and the visuospatial or “where” system, encoded by the “dorsal” occipito-parietal pathway (20).

In our paradigm, while VP represented a basic visual perceptual task (21, 22), VS combined both the skill to find the objects’ differences (21, 23) as well as the competence regarding line orientation and shape judgment (21, 24). Therefore, the comparison VS > VP allowed for the subtraction of visual stimulation, visuoperceptual analysis, and motor response, with aim to highlight the specific process underlying visuospatial judgment.

Our study shows that PD patients had a reduced activation of the right insula, left putamen, bilateral caudate (in particular in right hemisphere), and right hippocampus, compared to controls. Recent neuroimaging studies have highlighted the importance of various connections among these regions, specifically nigral-insular (25) and striatal-insular (26) connections, that are part of basal ganglia networks involved in attention and cognitive functions (27).

Our finding regarding the putamen can be interpreted on the pathophysiological basis of PD (28), according to the right-sided onset of disease in our patients. Similarly, the pivotal role of caudate in cognitive functions (29), in particular visual perception process (30), has been confirmed. The bilateral defect in caudate activation in PD patients is in agreement with recent findings on the right hemispheric dominance for visual perception. Moreover, results on the impairment of this region suggest its role in deciding ambiguous contexts (31).

The hypo-activation of the right insula is one of the most interesting findings of our study. Insula has been found to be involved in cognitive functions of PD patients (10), particularly in the presence of hallucinations (32). In addition, this region is presumed to play a salient role in attentional and complex processing in healthy subjects (33) and is characterized by long-range functional interactions with visual perception networks (34). Accordingly, the activation of the right insula in our healthy controls could denote a “switch” between attentional and visuospatial networks provoked by specific VS > VP comparison (35), while the relative hypo-activation in PD patients may suggest the initial loss of efficiency, anticipating the development of cognitive impairment and visual hallucincations.

Our finding that the right hippocampus in PD patients is less activated, compared to controls, confirms the involvement of this region in cognitive features in PD (1, 3, 31, 36, 37). It is known that hippocampus contributes to allocentric frame of reference, as part of the “Top–Down” visual processing system, that is minimally impaired in PD from previous studies (38). Recent studies have also pointed out a specific role for the right hippocampus in processing and storage of spatial information, encoded by neocortical–hippocampal loop in healthy subjects (39, 40). Thus, although deficits in hippocampal activation in PD have been usually considered as reduced efficiency in executive and attentional functions, our results, due to the specificity of the paradigm, suggest an association between the hippocampus and subclinical visuospatial dysfunction in PD, but might also be due to levodopa.

PD patients showed greater activations of right DLPFC and bilateral PPC, compared to controls. Both of these regions, together with frontal–striatal circuits, are known to be part of the “Top–Down” visual processing system, which is involved in the selection and organization of complex visual information (41). In particular, DLPFC and PPC specifically contribute to egocentric frame of reference, which was reported to be impaired in PD patients showing visual perceptual deficits in previous studies (38, 42). Moreover, both regions are part of a visual perception network detected as developmental shift from frontal–cingulate–striatal network in young adulthood (43). Thus, the over-activation observed in PD patients compared to age-matched controls could be considered a consequence of a neurodegenerative process underlying PD, but might also be due to levodopa.

Regarding the DLPFC alone, it has been hypothesized that it has a role in executive abilities and its involvement has been observed in PD patients with cognitive impairment (44), even in the non-demented stage (1). A reduction of DLPFC activation in PD patients has usually been attributed to altered striatal–frontal projections (45), but based on recent findings not in agreement with the traditional model, an underlying deficient interplay between the nigrostrial and mesocortical dopamine system has been suggested (46). Furthermore, a resting state fMRI study reported a parcelation of DLPFC in different subregions that mark a transition to visuospatial/sensorimotor networks (47). Accordingly, an event-related fMRI study evidenced subregions of DLPFC selectively encoding for positive and negative visual spatial priming (48). Our DLPFC greater activation could be considered compensatory in PD patients, through a continuous control performed by the “Top–Down” visual processing system, which was observed to be linked to visual working memory (49). We observed a similar response in PD patients learning of a novel complex motor task (50). This latter observation could be consistent with the suggested difficulty of PD patients to use sensory information for planning and executing complex or new tasks (51).

Several studies on healthy subjects have demonstrated that bilateral PPC is specialized in visual discrimination and localization (52, 53). Although the right dominance of parietal cortex for visual perceptual abilities has been confirmed (54), recent neuroimaging studies have suggested new interesting roles for the left parietal cortex, including alerting attention (55), memory retrieval (56), and multisensory visual-tactile integration through a “Top–Down” system (7, 57). Moreover, a compensatory activation of left PPC, in the case of reduced activity of right PPC after transcranial magnetic stimulation, has been recently observed (58). Being so, right PPC is thought to share dynamic connections with bi-hemispheric remote regions during exogenously cued visuospatial attention (59). PPC has also been reported to be part of the basal ganglia circuits, given that diverse evidence regarding nigro-parietal and striatal-parietal connections has been recently reported (7, 25, 26).

In our study, PD patients showed a greater activation of bilateral PPC, with a right predominance, compared to controls. This result has not been previously reported, except for a comparison between PD patients without hallucinations versus PD patients with hallucinations (60). Our finding can be explained by the specificity of the task performed and by the clinical aspects of our patients. In fact, all PD patients presented a short duration of disease, in absence of cognitive impairment and hallucinations, and, notably, were right-handed with right-sided onset of disease. It has been suggested that right-sided onset PD is characterized by impairment in local level processing of visual perception information, due to left frontal and parietal hemispheric deficits, which can be antagonized by the attentional network. Conversely, left-sided onset PD is characterized by abnormal global level processing of visual perception information, due to right parietal deficit, under all conditions (7, 30). Since our PD patients performed the tasks with the same accuracy of controls, we hypothesize that the greater activation of bilateral PPC could be necessary to overcome the initial impairment of the network. In particular, the relatively lesser signal in left PPC, ipsilateral to the onset of neuropathological process, could represent an incomplete compensatory activity for executive processing (45).

Our study had a number of limitations. First, because of the limited number of enrolled subjects and the magnetic field strength, the activation of some areas, such as the cerebellum, thalamus, and subthalamic nucleus (61), may not have been detected. Similarly, a functional connectivity mapping was not performed. It is possible that for this reason we were unable to detect the activation of the inferior frontal junction area and the precuneus, which are part of cognitive networks involving all areas and regions listed in our findings (62, 63). Finally, since PD patients were not tested in the ON drug condition, we did not perform the ON versus OFF comparison, which might yield changes specifically related to striatal dopamine depletion. It should be investigated in future studies.

## Conclusion

Our study analyzed the brain activations involved in salient aspects of visual perception in PD patients. Our findings suggest that the basal ganglia and limbic structure defects have a determining role in subclinical visuoperceptual and visuospatial impairments. Further investigations are needed to determine the roles of side of onset, disease duration, treatment, and exhaustive cognitive profile in the pathophysiology of visuospatial functioning in PD.

## Conflict of Interest Statement

The authors declare that the research was conducted in the absence of any commercial or financial relationships that could be construed as a potential conflict of interest.
